# Module-Based Association Analysis for Omics Data with Network Structure

**DOI:** 10.1371/journal.pone.0122309

**Published:** 2015-03-30

**Authors:** Zhi Wang, Arnab Maity, Chuhsing Kate Hsiao, Deepak Voora, Rima Kaddurah-Daouk, Jung-Ying Tzeng

**Affiliations:** 1 Bioinformatics Research Center, North Carolina State University, Raleigh, North Carolina, 27695, United States of America; 2 Department of Statistics, North Carolina State University, Raleigh, North Carolina, 27695, United States of America; 3 Institute of Epidemiology and Preventive Medicine, College of Public Health, National Taiwan University, Taipei, Taiwan; 4 Institute for Genome Sciences and Policy, Duke University, Durham, North Carolina, United States of America; 5 Department of Psychiatry and Behavioral Sciences, Duke University, Durham, North Carolina, United States of America; 6 Department of Statistics, National Cheng-Kung University, Taiwan, R.O.C; Indiana University Bloomington, UNITED STATES

## Abstract

Module-based analysis (MBA) aims to evaluate the effect of a group of biological elements sharing common features, such as SNPs in the same gene or metabolites in the same pathways, and has become an attractive alternative to traditional single bio-element approaches. Because bio-elements regulate and interact with each other as part of network, incorporating network structure information can more precisely model the biological effects, enhance the ability to detect true associations, and facilitate our understanding of the underlying biological mechanisms. How-ever, most MBA methods ignore the network structure information, which depicts the interaction and regulation relationship among basic functional units in biology system. We construct the con-nectivity kernel and the topology kernel to capture the relationship among bio-elements in a mod-ule, and use a kernel machine framework to evaluate the joint effect of bio-elements. Our proposed kernel machine approach directly incorporates network structure so to enhance the study effi-ciency; it can assess interactions among modules, account covariates, and is computational effi-cient. Through simulation studies and real data application, we demonstrate that the proposed network-based methods can have markedly better power than the approaches ignoring network information under a range of scenarios.

## Introduction

Module-based analysis (MBA) aims to evaluate the effect of a group of biological elements (bio-elements in short) sharing common features, such as SNPs in the same gene, co-expressed genes, or metabolites involved in the same pathways. A module can be constructed based on biological knowledge, e.g., pathway databases [1–3], or based on computational algorithms, e.g., clusters of correlated bio-elements [4–6]. Modules may serve as a more appropriate analyzing unit to understand the complex biological system because most cellular functions are carried out by groups of interactive bio-elements rather than individual ones [7]. MBA can increase the detectability and reproducibility of association findings because bio-elements tend to have moderate individual effects but significant aggregate effect. By assessing bio-element effects in a functional context, e.g., pathways and biological processes, MBA also improves the interpretability of findings and facilitates the construction of follow-up biological hypotheses. Finally, for exploratory “omics” studies, which usually begin with a full scan of a long list of candidate bio-elements, MBA provides a natural way to reduce the total number of tests, and hence relax the multiple-testing burdens and improve power.

Current approaches of MBA can be roughly classified into two major categories. The first type is the “meta”-based methods, which assess the module effect by integrating testing results of individual bio-elements, e.g., minimum p-value and Fisher’s combined test [8–9]. The second type is the “mega”-based methods, which jointly model the effect of all bio-elements in a module, such as principle component regression [10–11] and kernel machine regression [12–14]. Compared to the “meta”-based approaches, it is believed that “mega”-based methods can better capture the complex joint effect among bio-elements within a module.

Most of these current MBA approaches ignore network information in biological system [7,15]. Bio-elements are connected with and regulate each other as part of network. For example, genes and gene products regulate each other’s expressions and form a gene regulatory network. Proteins physically bind each other to carry out important functions in molecule processes, e.g., DNA replications, and form a protein-protein interaction network. Metabolites in cellular metabolism are modified through a series of biochemical reactions, which can be integrated into a metabolic network. Bio-elements in the same neighborhood of a network space tend to have similar biological functions. Therefore, incorporating network structure information can more precisely model the biological effects, enhance the ability to detect true associations, and facilitate our understanding of the underlying biological mechanisms [16].

In the content of gene expression analysis, many approaches have been developed to utilize network structure information. The methods formulate the identification of important bio-elements as a variable selection question and incorporate network structure by either specifying a network-constrained penalty function [17] or incorporating Markov random field priors [18–21]. These methods have concentrated on evaluating the effects of a single module and identifying the specific bio-elements that cause the module-level significance. We consider a different aspect of MBA—-our work focuses on evaluating the effects of multiple modules and investigating the interplay among them. We develop two kernel functions to capture the structural relationship among bio-elements within a module: the topology kernel and the connectivity kernel. The topology kernels based on the topological overlap matrix (TOM) [22], which describes the module structure while minimizes structural noises [23]. The connectivity kernel considers the connectivity of a node and controls a node’s contribution to the analysis based on the number of connections it has. We demonstrate that the proposed network-based methods can have markedly improved power over the approaches ignoring network information through simulation studies and a real-data analysis of pharmacometabolomics studies focused on aspirin.

## Materials and Methods

### Kernel machine regression model

Consider a sample with *n* subjects. Let *Y*_*i*_ represent the continuous trait value; *X*_*ℓi*_ *=* (*X*_*ℓi*1,_ *X*_*ℓi*2,…,_ *X*_*ℓiLℓ*_) be a vector containing values of the *L*_*ℓ*_ bio-elements in Module *ℓ*, *ℓ =* 1,2. Let *Z*_*i*_ *= Z*_*i*1_,*Z*_*i*2_,…,*Z*_*iQ*_) be a *Q×*1 vector containing covariates that are not included in either *X*_*1i*_ or *X*_*2i*_. We use the following semiparametric regression to model the relationship between the traits and the bio-elements in Module 1 and Module 2, which includes the module main effects, *h*_1_(·) and *h*_2_(·), and their interaction effect, *h*_12_(·), adjusting for the covariates *Z*_*i*_:

$$Y_{i}=Z_{i}^{T}\beta +h_{1}\left(X_{1i}\right)+h_{2}\left(X_{2i}\right)+h_{12}\left(X_{1i},X_{2i}\right)+\varepsilon_{i},$$ (1)

where *β* is a *Q×*1 vector of regression coefficients describing the effects of the covariates *Z*_*i*_, and *ε*_*i*_’s are independent random errors that follow a *N*(0,σ) distribution. In Model (1), functions h_*_(·)’*s* are the primary interests because they fully specify the relationship between bio-elements and trait. Under kernel machine framework, we assume that the nonparametric function h_*_(·) lies in a function space, $H_{K_{*}},$ generated by a positive definite kernel function K_*_(^.^,^.^). According to the Mercer’s theorem [24], h_*_(·) can be represented as the primal representation, $h_{*}\left(X_{i}\right)\;=\;\sum_{j\;=\;1}^{J}\phi_{j}\left(X_{i}\right)\eta_{j},\;$ where *ϕ*_*j*_(*X*_*i*_),*j* = 1,…,*j*, is a set of basis functions specified by K_*_(·,·). Equivalently, h_*_(·) can also be represented as the dual representation, $h_{*}\left(X_{i}\right)\;=\;\sum_{i^{'}\;=\;1}^{n}K_{*}\left(X_{i^{'}},\;X_{i}\right)\alpha_{i^{'}}\;$and *α*_*i*_ *’* are unknown parameters. Because h_*_(·) is fully defined by the kernel functions, by choosing different kernel functions, we can specify different bases and corresponding models to model module effects. Specifying h_*_(·) via the dual representation is more convenient than specifying it via the primal representation because explicit basis functions or features might be complicated. Many kernel functions have been constructed and are commonly used, e.g., the linear kernel function, given by $K_{*}\left(X_{i},X_{i^{'}}\right)\;=\;X_{i}^{T}X_{i^{'}}$, the second order polynomial kernel function, given by $K_{*}\left(X_{i},X_{i^{'}}\right)\;=\;{\left(1+X_{i}^{T}X_{i^{'}}\right)}^{2}$, and the Gaussian kernel, given by $K_{*}\left(X_{i},X_{i^{'}}\right)\;=\;exp\left\{\sum_{j\;=\;1}^{M}{\left(x_{ij}-x_{i^{'}j}\right)}^{2}/d\right\}$, where *d* is a tuning parameter.

### Kernel functions integrating network information

One appealing feature of kernel machine framework is that it allows for the inclusion of prior information in the kernel function to assist in the evaluation of module effects. In this paper, we introduce two network-based kernels to incorporate network information: the *topology kernel* and the *connectivity kernel*. Both kernels require a known network structure to begin with. Such network structure, typically summarized in the adjacency matrix [25], can be obtained from existing biological knowledge [2] or be constructed from the data (e.g., co-expressed gene modules). Given a network structure, the adjacency matrix is defined as *A* = [*A*_*ll’*_], where *A*_*ll’*_ = 1 if nodes *l* and *l’* are connected in the network, and *A*_*ll’*_ = 0 otherwise including *l = l’*. When network structure is unknown, there are many methods that can be used to estimate the adjacency matrix. These methods can be roughly classified into four categories [26]: (a) pairwise correlation methods, e.g., WGCNA[5,27]; (b) partial correlation methods, e.g., GeneNet [28]; (c) information theory methods, e.g., ARACNE [29–30] and TINGE [31]; and (d) Bayesian Network, e.g., [32–34]. Briefly speaking, Type (a) does not distinguish direct and indirect correlations among modules but is easy to compute. Type (b) uses Gaussian graphic model to capture the multivariate dependence among genes and builds the adjacency matrix only with direct correlations. Type (c), information-based method, can identify both linear and non-linear (direct) dependencies while model-based methods tend to focus on linear correlations. Type (d) can better handle noises in the data but tends to be more computationally intensive.

### The topology kernel function *K* ^*Top*^(*X*_*i*_,*X*_*i*_)

We construct the topology kernels based on the topological overlap matrix (TOM) [22], which can be computed from the adjacency matrix, *A*, as given in Equation (2) below. TOM is considered as an alternative to the adjacency matrix to minimize structural noises when describing the module structure [23]; empirical studies [22,35–37] have shown that nodes having a higher topological overlap are more likely to belong to the same functional class. Given matrix *A*, the corresponding TOM, denoted by *T*≡[*T*_*ll’*_], is

$$T_{ll^{\prime }}=\{\begin{array}{cc} \frac{L_{ll^{\prime }}+A_{ll^{\prime }}}{min\left\{k_{l},k_{l^{\prime }}\right\}-A_{ll^{\prime }}+1} & forl\neq l^{\prime }\\ 1 & forl=l^{\prime } \end{array},$$ (2)

where *L*_*ll’*_ = Σ_*u≠l*_,_*l’*_ *A*_*lu*_ *A*_*l’u*_, which is the number of neighbors shared between nodes *l* and *l’*; *A*_*ll’*_ indicates if nodes *l* and *l’* are directly connected to each other;*k*_*l*_ *=* Σ_*u≠l*_,_*l’*_ *A*_*lu*_, which quantifies the number of direct neighbors (edges) that node *l* has. From Equation (2), we can see that, in contrast to adjacency matrices, TOM describes the network structure using both *L*_*ll’*_ and *A*_*ll’*_, that is, TOM measures the node relationship not only based on the pair of nodes themselves but also their relationship to all other nodes in the network. In other words, for nodes *l* and *l’* that are not directly connected in a network (e.g.,*A*_*ll’*_ = 0), they are still considered as “closely connected” in terms of high topological overlaps as long as they share common neighbors (e.g.,*L*_*ll’*_ *≠*0). The denominator of Equation (2) is a normalizing factor so that the range of *T*_*ll’*_ is between 0 and 1 because *A*_*ll’*_≤1 and *L*_*ll’*_≤min(*k*_*l*_,*k*_*l’*_)-*A*_*ll’*_ by Yip and and Horvath [23].

We incorporate the TOM into the topology kernel by $K^{Top}\left(X_{i},X_{i^{'}}\right)\equiv X_{i}^{T}{TX}_{i^{'}}.$ To fix the idea, here we consider the linear kernel but the same idea can be extend to other kernel such as polynomial kernels. The topology kernel encourages similar effects for those nodes “close” in a network. The smoothing effect can been more clearly seen from a Bayesian perspective as discussed in the conclusion section.

### The connectivity kernel function *K* ^*Con*^(*X*_*i*_,*X*_*i*_)

Alternatively, we can incorporate different type of network information from the topological overlap. Specifically, the connectivity kernel, defined as $K^{Con}(X_{i},X_{i^{'}})\;=\;X_{i}^{T}{WX}_{i^{'}}$ where *W* is a diagonal matrix with $W_{ll}\;=\;\sum_{l^{'}\neq l}^{}T_{ll^{'}}$, considers the connectivity of a node and controls a node’s contribution to the analysis based on the number of connections it has, i.e., $\sum_{l^{'}\neq l}^{}T_{ll^{'}}$ for node *l*. The functional and structural importance of hub nodes have been established in the literature: Removing hub nodes from the network would severely alter network structure [38] and impact the network function and organismal fitness [39–41]. The connectivity kernel intends to upweight hub nodes so as to reflect the fact that hub nodes tend to play a more substantial role than non-hub nodes in a network [42]. For example, it is found that in the yeast protein–protein interaction networks, hubs are more likely to be essential and conserved than non-hub proteins [7,43].

Here we construct our network kernels based on the TOM. When needed, one can replace TOM by the adjacency matrix or even correlation matrix. Nevertheless, we expect several advantages for using TOM. TOM has been empirically demonstrated to be a meaningful measure on interconnectedness in real biological networks [27,44]. In addition, compared to the adjacency matrix, the TOM is more tolerant to errors caused by spurious or missing edges between two nodes because TOM considers the neighboring structure of the two nodes in addition to their direct connectivity. The edges of a network cannot always be precisely determined due to too noisy or incomplete network information, especially if edges are obtained from relevance network. The adjacency matrix, which is constructed based on direct connection, is noted to be sensitive to noises and lead to wrong network inference [27].

### Kernel functions for interaction effects

To model between-module interaction effect, we construct an interaction kernel by taking the element-wise product of the main-effect kernels:

$$K_{12}\left(\left(X_{1i},X_{2i}\right),\left(X_{{1i}^{'}},X_{{2i}^{'}}\right)\right)\;=\;K_{1}\left(X_{1i},X_{{1i}^{'}}\right)K_{2}\left(X_{2i},X_{{2i}^{'}}\right),$$

where *K*_*1*_(·,·) and *K*_*2*_(·,·) are kernels used for Modules 1 and 2, respectively. If other kernels, such as polynomial kernels, were used, one would need to remove the constant term in these kernels as suggested in Wang et al. [45] so as to avoid false positive and false negative findings that are caused by including duplicated main effect term in the interaction kernel.

### Testing module effects

We developed two score-based tests under Model (1) to assess module effects. The first is the interaction test for assessing module-module interaction, i.e., to test $H_{0}^{X_{1}*X_{2}}:h_{12}\left(∙\right)\;=\;0$. The second is the conditional test for assessing the effect of a certain module adjusting for the other module, i.e., to test $H_{0}^{X_{1}|X_{2}}:h_{1}\left(∙\right)\;=\;0$ without constraining *h*_2_(·)but under the constraint of *h*_12_(·) = 0. The test for $H_{0}^{X_{2}|X_{1}}:h_{2}\left(∙\right)\;=\;0$ can be defined by the same manner. To test these hypotheses, we consider the following mixed model representation of kernel machine regression (1) as did in Liu et al. [13] and Wang et al. [45]:

$$Y=Z\beta +h_{1}+h_{2}+h_{12}+\varepsilon ,$$ (3)

where *Y* ^*T*^ *=* (*Y*_1_,…,*Y*_*n*_) $h_{l}^{T}\;=\;\left(h_{l1},\cdots ,h_{ln}\;\right)\sim N\left(0,\tau_{l}K_{l}\right)$ with *h*_*ℓi*_ being the effect of Module ℓ for subject i, ℓ = 1,2, $h_{12}^{T}\;=\;\left(h_{12,1},\cdots ,h_{12,n}\right)\sim$ N(0,*τ*_12_ *K*_12_) with *h*_12,*i*_ being the interaction effect of Modules 1 and 2 for subject *i* and *ε* = (*ε*_*1*_,…,*ε*_*n*_)~*N*(0,σ*l*_*n*_) Consequently, testing *H*_0_:h_*_(·) under kernel machine regression (1) is equivalent to testing *H*_0_:τ_*_ *=* 0 vs. *H*_*A*_:τ_*_ *>* 0 under the linear mixed model (3).

We derive score tests for the interaction test and the conditional test based on the restricted maximum likelihood (REML) of the Model (3); the derivations are given in S1 Appendix. Specifically, the test statistic for the interaction test ($T_{X_{1}*X_{2}})$, the conditional test of Module 1 ($T_{X_{1}|X_{2}}$) and the conditional test of Module 2 ($T_{X_{2}|X_{1}}$) are given as follows.

$$T_{X_{1}*X_{2}}\;=\;\frac{Y^{T}P_{12}K_{12}P_{12}Y}{2}\;{\left.\right|}_{\tau_{12}\;=\;0,\tau_{1}\;=\;{{\hat{\tau }}_{1},\;\tau }_{2}\;=\;{\hat{\tau }}_{2},\;\sigma \;=\;{\hat{\sigma }}_{X_{1}{*X}_{2}}\;},$$

$$T_{X_{1}|X_{2}}\;=\;\frac{Y^{T}P_{1}K_{1}P_{1}Y}{2}\;{\left.\right|}_{\tau_{12}\;=\;0,\tau_{1}\;=\;{0,\;\tau }_{2}\;=\;\tilde{\tau_{2}},\;\sigma \;=\;{\tilde{\sigma }}_{X_{1}{|X}_{2}}\;}, and$$

$$T_{X_{2}|X_{1}}\;=\;\frac{Y^{T}P_{2}K_{2}P_{2}Y}{2}\;{\left.\right|}_{\tau_{12}\;=\;0,\tau_{1}\;=\;{\tilde{\tau_{1}},\;\tau }_{2}\;=\;0,\;\sigma \;=\;{\tilde{\sigma }}_{X_{2}{|X}_{1}}\;},$$

Where *Y* ^*T*^ *=* (*Y*_1_,…,*Y*_*n*_), $P_{t}\;=\;V_{t}^{-1}-V_{t}^{-1}Z{\left(Z^{T}V_{t}^{-1}Z\right)}^{-1}Z^{T}V_{t}^{-1}\;$for *t =* {12,1,2}, *K*_*t*_ *= K*_*t*_(·,·)for *t*∈ *=* {12,1,2}, *V*_12_ = *τ*_*1*_ *K*_1_+*τ*_*2*_ *K*_2_+σ*l*_*n*_,*V*_1_ = *τ*_*2*_ *K*_2_+σ*l*_*n*_ and *V*_2_ = *τ*_*1*_ *K*_1_+σ*l*_*n*_. The estimates $\hat{{(\tau }_{1}},\hat{\tau_{2},\;}{\hat{\sigma }}_{X_{1}{*X}_{2}},\;\tilde{\tau_{2}},\;{\tilde{\sigma }}_{X_{1}{|X}_{2}},$ $\tilde{\tau_{1}},\;{\tilde{\sigma }}_{X_{2}{|X}_{1}}$) are obtained from the EM algorithms as described in the Appendix. We also show in the Appendix that these test statistics asymptotically follow a weighted chi-squared distribution [46–47], and the corresponding p-values can be obtained by moment matching approaches [48].

## Results

### Simulation studies

We conducted two simulation studies to evaluate the performance of network-based approaches. Simulation I considered modules with scale-free structures and Simulation II considered modules with non-scale-free structures. In each simulation, we compared the kernel machine regression with network-based kernels (i.e., topology kernel and connectivity kernel) to the same approach ignoring the network information (i.e., unstructured kernel).

#### Simulation I: Scale-free modules

**Fig 1:**
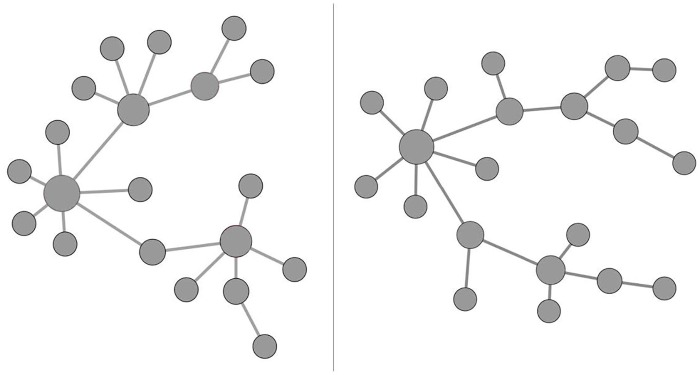
Modules with sale-free structures used in Simulation I. The left panel is Module 1 and the right panel is Module 2. These modules were simulated based on Barabási–Albert model using package *igraph* in R.

We generated two 20-node modules with scale-free structure based on Barabási–Albert model [49] and the network structures of the two modules are given in Fig. 1. The scale free structures have three well-known features. First, the connectivity of nodes follows power law. Specifically, define *k* the number of edges that a node have. The probability distribution of *k* has the form of *p*(*k*)∝*k* ^*-δ*^ with a certain constant *δ* (a network parameter). That is, the probability of observing a node with *k* edges decreases exponentially as *k* increases. Second, nodes with top connections (i.e., hub nodes) are assumed to play specific roles. Finally, network with scale-free structures are more error tolerant, i.e., random loss of a node in a scale-free network is less destructive than in a random network.

#### Simulation II: Non-scale-free modules

**Fig 2:**
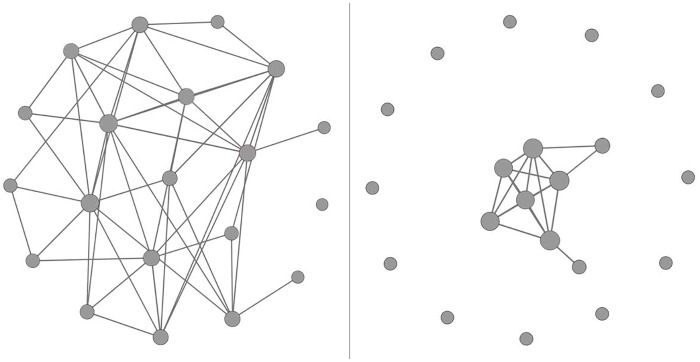
Modules with non-scale-free structures used in Simulation II. The left panel is Module 1 and the right panel is Module 2.

Although the scale-free structure is the most common network structure in real practice, in reality, it is also possible to obtain modules that do not have such ideal structure due to several reasons. First, sub-networks sampled from a scale-free network are not necessarily scale free [50]. In addition, investigators tend to profile hubs instead of the entire network at the first place in order to reduce the cost. Finally, investigators may not be able to observe the complete network and meanwhile include many irrelevant nodes in the study because of limited knowledge on the network. In Simulation II, we considered two causal modules with structures presented in Fig. 2. Module 1 consisted of 20 nodes that were highly connected, while Module 2 consisted of 20 nodes that were loosely connected. Both modules were subsets of a large scale-free module containing 100 nodes. Module 1 was obtained by taking the top 20 nodes that had the most connections; Module 2 was formed by taking the bottom 20 nodes that had the fewest connections.

Given the causal modules with certain structures, we followed the simulation design used in Monni and Li [20] to generate the values of bio-elements and responses in Simulation I and Simulation II. For subject *i*, let *Y*_*i*_ represent the continuous trait value; let *X*_1*i*_ and *X*_2*i*_ be the design vectors of the nodes in Module 1 and Module 2, respectively. First, we generated *X*_1*i*_ (and *X*_2*i*_) from a multivariate normal distribution with pairwise correlation Cor(*X*_s_,*X*_*t*_) = *G*_*st*_ */*2, where *G*_*st*_ = 1 if nodes *s* and *t* are directly connected in the module and 0 otherwise. We then selected the causal nodes under two scenarios. In the first scenario, we deliberately set hub nodes as causal, i.e., assigning the top 4 nodes with most connections in each module. In the second scenario, we randomly selected *C* nodes from each module as causal with *C* = 4, 10 and 16. Such design is to mimic the scenario that changes in the network occur randomly rather than initiated by hubs to influence the response, presumably due to mutations or environmental factors. Next, we generated response value *Y*_*i*_ from a Normal distribution with mean *μ*_*i*_ and variance *ζ*. We let $\mu_{i}\;=\;{\gamma_{1}\times \tilde{X}}_{1i}^{T}\beta_{1}+{\gamma_{2}\times \tilde{X}}_{2i}^{T}\beta_{2}+{\gamma_{12}\times \tilde{X}}_{12i}^{T}\beta_{12}$, where ${\tilde{X}}_{li},\;l\;=\;1,2$ is the design vector of the causal nodes in Module ℓ for subject *i*, ${\tilde{X}}_{12i}$ is the design vector including all pairwise interactions between ${\tilde{X}}_{1i}\;and\;{\tilde{X}}_{2i}$, and effect size *β*_*ℓ*_’s were randomly determined from the uniform distribution with interval *l =* [-0.2, -0.05]⋃[0.05,0.2]. We adjusted the values of the variance *ζ* to reflect different magnitudes of noise-to-signal ratios. Specifically, values of *ζ* were determined so that the *R* ^2^ values explained by *μ*_*i*_ could yield power within a reasonable range. For type I error rate analysis, we set (*γ*_1_,*γ*_2_,*γ*_3_) = (0,0,0) and performed 1000 replications. For the power analysis, we performed 250 replications and the values of (*γ*_1_,*γ*_2_,*γ*_3_) was set to be (0,0,1) for the interaction test, (1,0,0) for the conditional tests of Module 1, and (0,1,0) for the conditional test of Module 2. We simulated 1000 individuals per replication.

### Type I error analysis of Simulations I and II

In both simulations, the type I error rates were around the 0.05 nominal level for all kernel functions under all scenarios (Table 1). The results suggest the validity of the asymptotic distributions for the proposed statistics. It also assured the validity of our KM regression and the legitimacy of power comparisons presented next.

**Table 1:** Type I error rates averaged over 1000 replicate data sets.

*Hull Hypothesis being Tested*			
	M1*M2	M1|M2	M2|M1
***Simulation 1*** *			
Topology^†^	0.047	0.043	0.050
Connectivity	0.038	0.054	0.051
Unstructured	0.042	0.050	0.048
***Simulation 2***			
Topology	0.045	0.050	0.044
Connectivity	0.042	0.049	0.050
Unstructured	0.052	0.044	0.040

* For details of simulation 1 and 2 see simulation section.

^†^ Topology = Topology Kernel; Connectivity = Connectivity Kernel; Unstructured = Linear Kernel. For details of various kernels see method section.

### Power analysis in Simulation I (scale-free modules)

**Fig 3:**
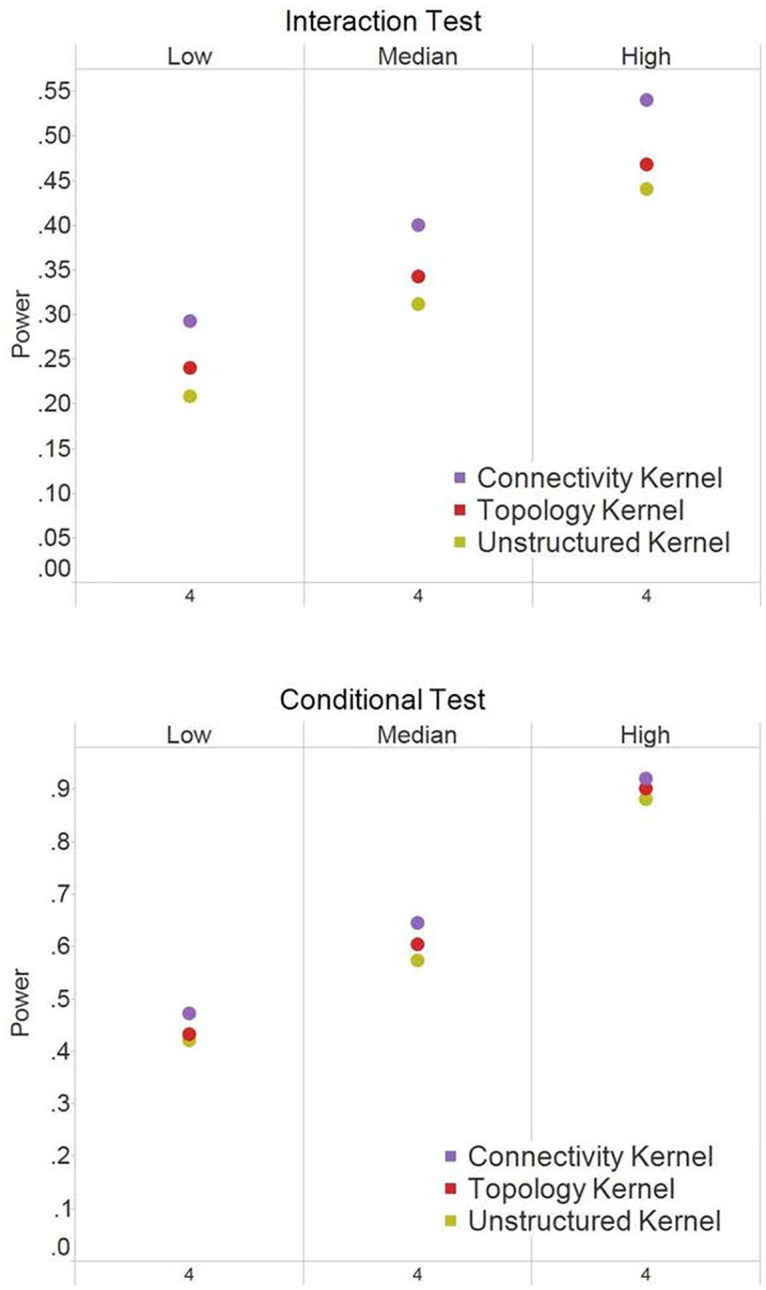
Power results for Simulation I (scale-free structure) when causal nodes are hub nodes. The power at α = 0.05 were based on 250 simulation replications for the interaction test and the conditional test of Module 1. The X-axis indicates the number of causal nodes out of the 20 nodes in a module. The three panels under each test, i.e., *Low*, *Median*, and *High*, indicate the level of the R^2^ explained by the module effects.

Under the scenario of causal hub nodes (Fig. 3), the connectivity kernel performed the best, followed by the topology kernel and then the unstructured kernel. The pattern held across different *R* ^2^ values and for both interaction test and the conditional test. As expected, this is because the causal nodes, which have high connectivity, were most substantially up-weighted by the connectivity kernel than the other two kernels. Here we only show one of conditional test results (conditional test of Module 1). Similar conclusions hold for both conditional tests because Modules 1 and 2 have similar scale-free structure.

**Fig 4:**
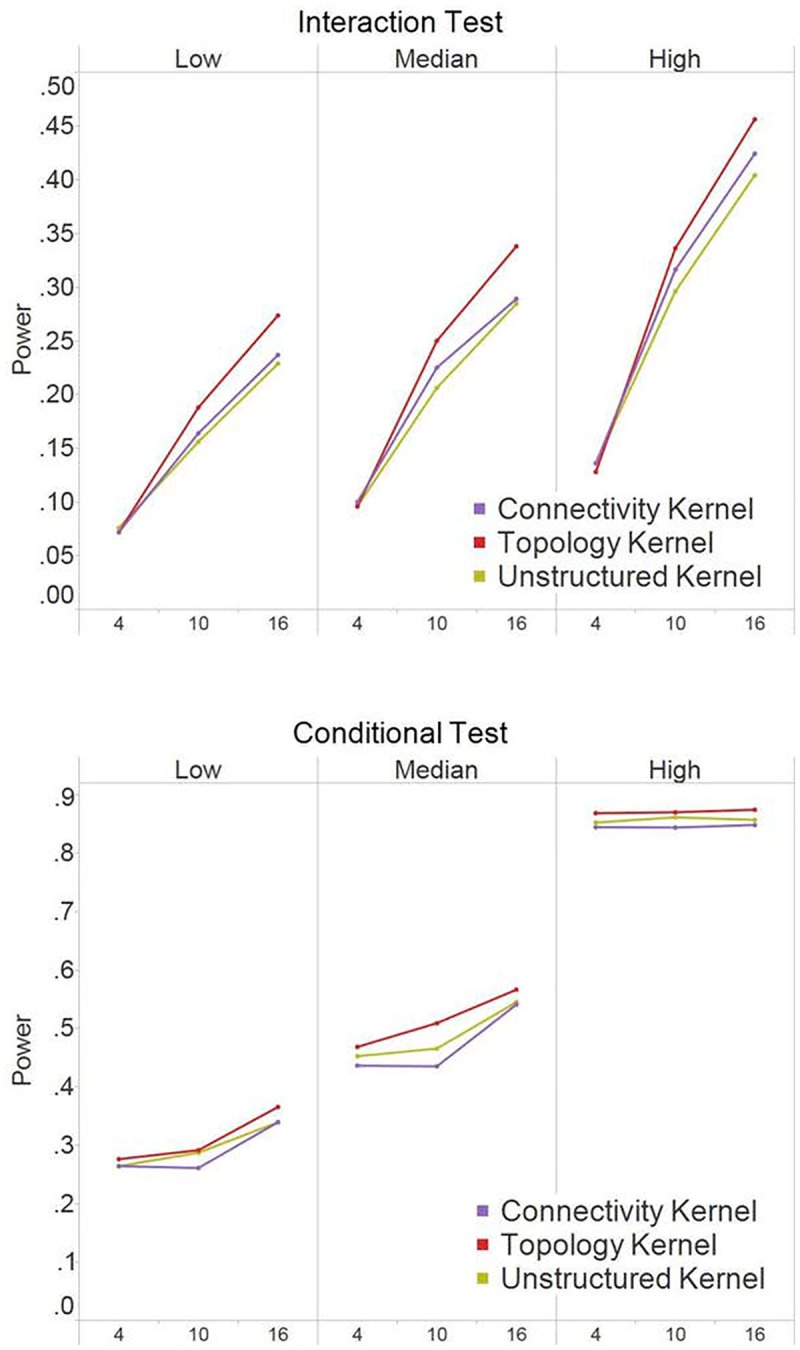
Power results for simulation I (scale-free structure) when causal nodes are random nodes. The power at α = 0.05 were based on 250 simulation replications for the interaction test and the conditional test of Module 1. The X-axis indicates the number of causal nodes out of the 20 nodes in a module. The three panels under each test, i.e., *Low*, *Median*, and *High*, indicate the level of the R^2^ explained by the module effects.

Under the scenario when the causal nodes were randomly selected (Fig. 4), the topology kernel had top performance across all scenarios including different number of causal nods, different magnitude of *R* ^2^, different type of tests (interaction vs. conditional tests). Because causal nodes were randomly selected, they are more likely to be secondary nodes which are the majority in the scale-free structure. Among the three kernel methods, the topology kernel can best capture the signals from secondary nodes. The power gain by the topology kernel increased when the number of causal nodes increases. We also observed that incorporating connectivity information did not always help in improving power. For interaction test, the connectivity kernel had comparable power to the unstructured kernel, while for the conditional test, the connectivity kernels had comparable or worse performances compared to the unstructured kernel. This is likely because the connectivity kernel overly weighted hub nodes and missed the signals from non-hub nodes.

### Power analysis in Simulation II (non-scale-free modules)

**Fig 5:**
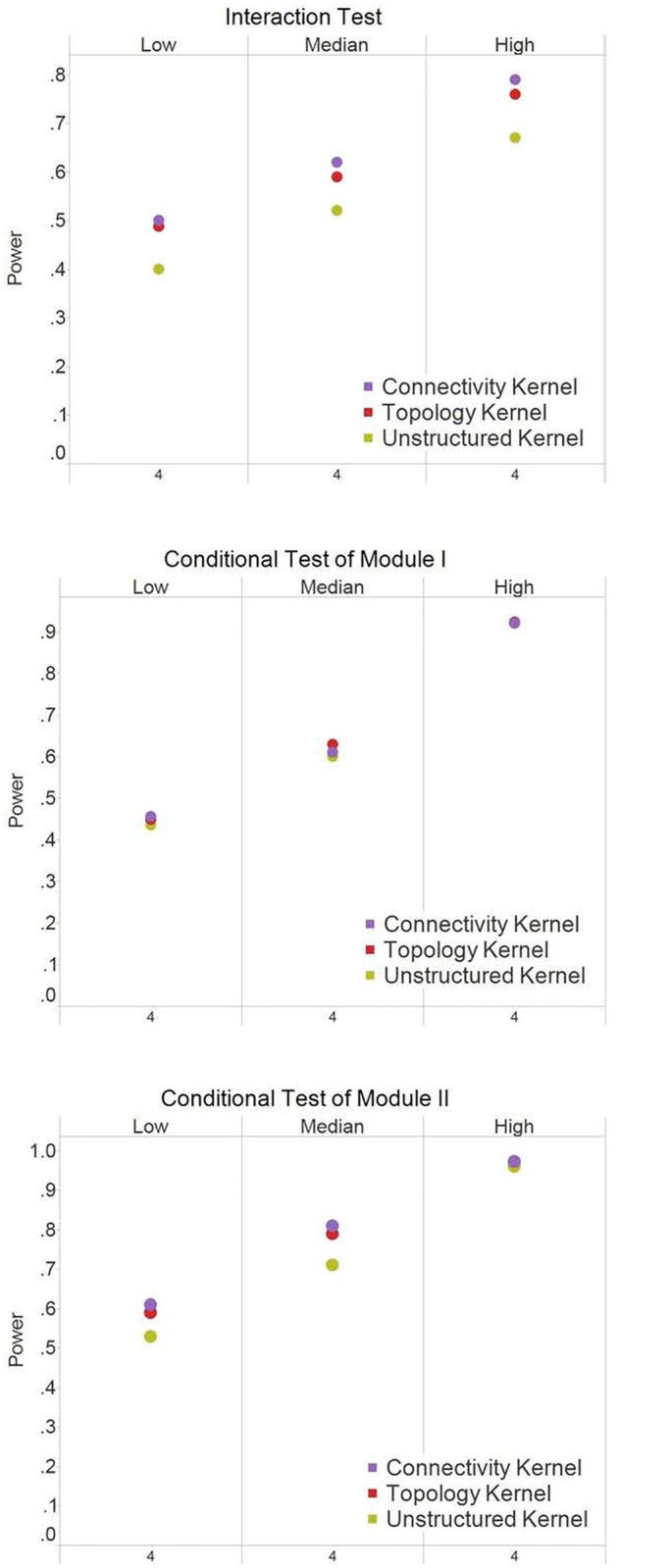
Power results for simulation II (non-scale-free structure) when causal nodes are hub nodes. The power at α = 0.05 were based on 250 simulation replications for the interaction test and the conditional tests. The X-axis indicates the number of causal nodes out of the 20 nodes in a module. The three panels under each test, i.e., *Low*, *Median*, and *High*, indicate the level of the R^2^ explained by the module effects.

Similar to what is observed in Fig. 3, when the causal nodes were hubs (Fig. 5), the connectivity kernel often had the best performance among all three tests with different levels of *R* ^2^. However, the amount of power gain by the connectivity kernel was not as substantial as in Simulation I of scare-free modules. This is because one of the modules (i.e., Module 1 with highly connected nodes) had similar numbers of edges for hub nodes and non-hub nodes. In addition, because the numbers of edges for hub nodes were much higher than non-hub nodes in Module 2 while being similar in Module 1, we see the three tests performed similarly only in the conditional test of Module 1.

**Fig 6:**
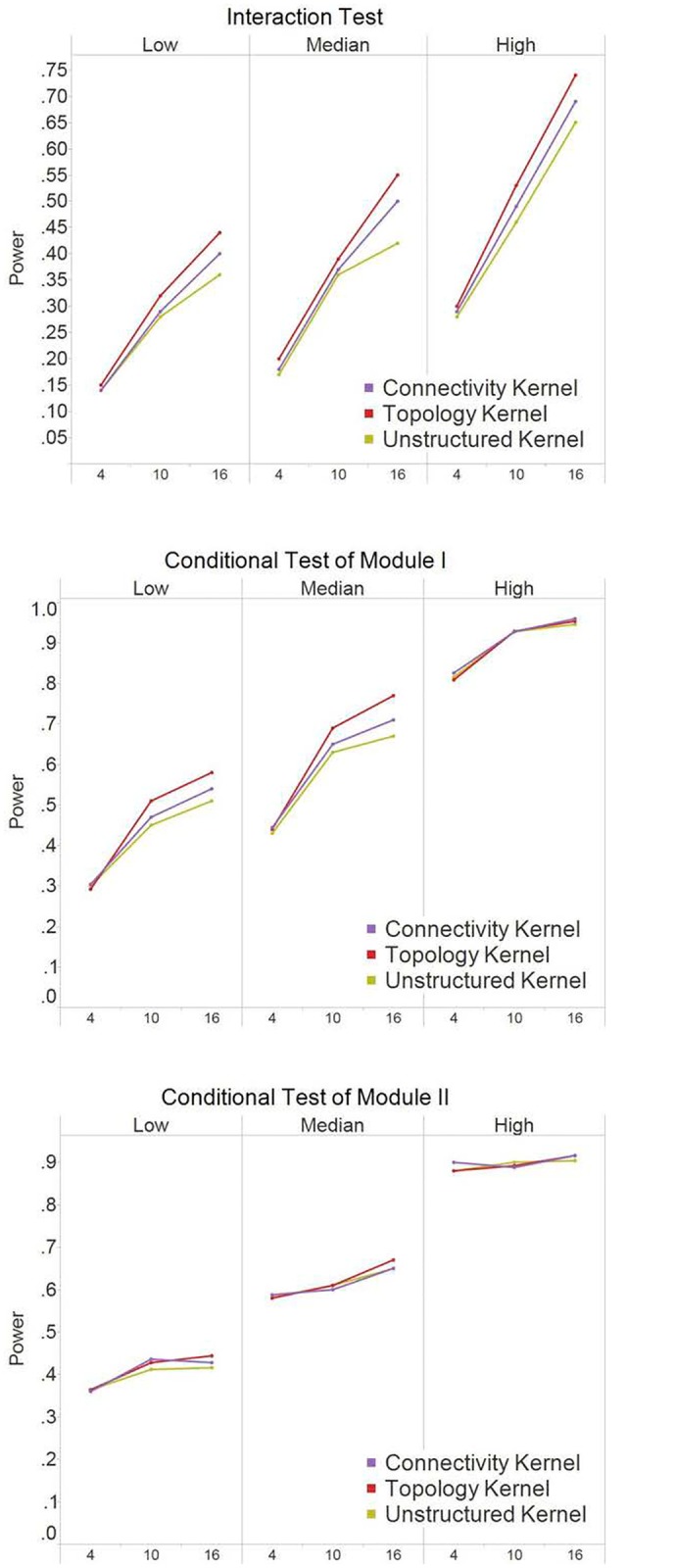
Power results for simulation II (non-scale-free structure) when causal nodes are random nodes. The power at α = 0.05 were based on 250 simulation replications for the interaction test and the conditional tests. The X-axis indicates the number of causal nodes out of the 20 nodes in a module. The three panels under each test, i.e., *Low*, *Median*, and *High*, indicate the level of the R^2^ explained by the module effects.

When the causal nodes were randomly selected (Fig. 6), the topology kernel had the best performance among the three kernels for the interaction tests and the conditional test of Module 1. The results are similar to Fig. 4. The power gain by the topology kernel increased when the number of causal nodes increases. When only a few nodes were selected as causal in the non-scale-free modules, most causal nodes were likely to have similar structure background with non-causal nodes (e.g., being isolated or having similar topology and connectivity level). Consequently, incorporating network information did not aid much in power, though it did not hurt the performance either. By the same reasons, we also observed that, in the conditional test of Module 2, the three kernels performed comparably (as no obvious structural difference among causal and non-causal nodes when the causal nodes were randomly selected from sparsely connected Module 2).

### Real data applications

We use proposed kernel machine regression method to analyze plasma metabolomics data collected through the Pharmacometabolomics Research Network. Briefly, healthy volunteers were exposed to 325mg/day aspirin for four weeks as previously described [51]. Plasma from before and after aspirin exposure was examined by GC-TOF Mass Spectrometry as described in Wikoff et al. [52]. Aspirin is the antiplatelet agent of choice used for the prevention of myocardial infarction (MI) and stroke [53]. However, interpersonal variation has been observed in response to aspirin. About 10~20% people develop MI and stroke despite aspirin use, which raises the possibility of a failure of aspirin to fully inhibit platelet function [54]. Recently the Pharmacometabolomics Research Network has published a series of papers demonstrating that metabolic profile of a patient at baseline and prior to treatment informs about treatment outcome [55–58]. In addition, trajectory of metabolic changes early in the course of treatment can provide additional valuable information about drug response phenotypes. Therefore, the goal of this study is to study the association between metabolic phenotypes and the response to aspirin.

The aspirin metabolomics data was gathered from 53 healthy volunteers; for each individual, 403 metabolites were measured including 151 knowns and 252 unknowns. The drug response, platelet aggregation inhibition, is quantified by a composite score which is the first principle component of a series of measurements of platelet aggregation and has been described previously [59]. These measurements are the area under the aggregometry curve induced by collagen, epineprhrine, and ADP at different concentrations and the PFA100 (collagen/epinephrine) closure time. Preliminary study was performed using single-metabolite analyses which assess metabolic effects on drug response one metabolite at a time. Because metabolites do not function in isolation, module-based metabolite analysis may serve a more powerful alternative to identify the metabolic groups that influence the drug responses.

To illustrate the utility of the proposed methods, we selected candidate modules using the procedure as follows. First, we used the weighted correlation network analysis (WGCNA) [5] to find modules of highly correlated metabolites. We then performed an over-representation analysis (ORA) on each module to identify modules that were enriched with “promising” metabolites (e.g., metabolites with p-values less than 0.2 from the single-metabolite analyses). Although modules can also be constructed by knowledge-based approaches such as KEGG, forming module based on correlation pattern allowed us to incorporate unknown metabolites in the analysis.

We performed two sets of analyses: one focused on evaluating the baseline metabolic measurements vs. baseline measures of platelet aggregation (referred to as the *baseline* analysis), and the other focused on the change of metabolic measurements vs. the change in measures of platelet aggregation (referred to as the *difference* analysis). In the *baseline* analysis (Table 2), there were two candidate modules (referred to as Module 1 and Module 2) identified from the module discovery procedure mentioned above. In the kernel machine analysis, we started with the Interaction test to assess the interactions between these two modules using the proposed kernels that incorporating network information, i.e., the connectivity kernel and the topology kernel. Both analyses indicated significant interactions between these two modules (the p-values for connectivity kernel and topology kernel are 0.019 and 0.013, respectively). To compare, we repeated the same analysis using the unstructured kernel, and the p value is not significant (0.055). Because of the significant findings of the interaction tests, we do not proceed further with the conditional main effect tests in the baseline analyses.

**Table 2:** Testing results from the *baseline analysis* of the Aspirin Data.

Kernel	M1|M2*	M2|M1	M1*M2
Connectivity	NA	NA	0.019
Topology	NA	NA	0.013
Unstructured	0.17	0.62	0.055

* M1|M2:conditional test of Module 1; M2|M1: conditional test of Module 2; M1*M2: interaction test between Module 1 and Module2.

In the *difference* analysis (Table 3), there were also two modules (referred to as Module 3 and Module 4) identified from module discovery procedure. We then started with the interaction analysis using the network-structured kernels. The interaction test was not significant for both kernels. We hence proceeded with the conditional tests and found that both modules are significant. When using the unstructured kernel, the interaction effect was not significant either, and there was only one module with significant conditional effect on platelet aggregation (Module 3 given Module 4; p-value 0.032).

**Table 3:** Testing results from the *differential analysis* of the Aspirin Data.

Kernel	M3|M4*	M3|M4	M3*M4
Connectivity	0.030	0.039	0.38
Topology	0.023	0.042	0.58
Unstructured	0.032	0.052	0.40

* *M3|M4*: *conditional test of Module 3; M4|M3*: conditional test of Module 4; M3*M4: *interaction test between Module 3 and Module 4*.

To gain biological insights of the results, we mapped the known metabolites in the significant modules to the KEGG pathway. We used KEGG Mapper to see if any pathways are enriched by the known metabolites in Module 1 to Module 4. The results indicated that that the biosynthesis of fatty acid pathway is over-represented by Module 3 and Module 4. Specifically, in these fatty acids, arachidonic acid is known to be a precursor in the production of thromboxane A2 (TXA2), which triggers reaction that lead to platelet aggregation. Aspirin acts as antiplatelet agent by inhibiting the COX1 enzyme, which is a key enzyme in TXA2 generation. This finding suggests a potential relationship between biosynthesis of fatty acids pathway and aspirin’s effects on platelets. Studies [60–61] show that there is interference between fatty acids and platelet inhibition by aspirin.

## Discussion and Conclusions

Module-based analysis has emerged as a powerful and flexible approach for studying the relationship between bio-elements and phenotypes [12–13,25,62]. However, most of these methods ignore the network structure information, which depicts the interaction and regulation relationship among basic functional units in biology system. Incorporating network information can aid with association detection and uncover underlining biological features. In this work, we proposed a KM approach that directly incorporates network structure to evaluate the joint effect of bio-elements. Specifically, we constructed the connectivity kernel and the topology kernel to capture the relationship among bio-elements in a module. The simulation studies and real data application suggest that our proposed network-based methods can have markedly better power than the approaches ignoring network information. The R code of the proposed tests is available to download at http://www4.stat.ncsu.edu/~jytzeng/Software/NetworkKernel/.

Our network KM procedure also has a Bayesian interpretation. Consider a simplified model with only one module effect: *Y* = *h+*ϵ. Further assume that a linear model is used to model the bio-element-set effect, i.e., *h = Xβ*. Then the proposed KM model with *K*_*T*_ = *XTX* ^*T*^, which is equivalent to *h~N*(0,*τK*_*T*_), can be viewed as imposing a prior on the coefficient *β* with *β~N*(0,*τT*). In other words, by incorporating the structure information, we encourage bio-elements nearer in the network space to share similar effects. The smoothing according to network topology also helps to stabilize the inference especially when the network is large. Finally, the topology structure is only included through prior information, which will guide, rather than force, the effect smoothing when the data are consistent with the prior information.

From our simulation results, we observed that the unstructured kernel tends have the lowest power among the three kernels (i.e., connectivity, topology and unstructured). In the simulations, for those scenarios where the causal genes are randomly selected from hub genes, the connectivity kernel would be a more "correct" kernel than the topological kernel. (In contrast, in those scenarios with the causal genes from non-hub genes, the topological kernel would more "correct" than the connectivity kernel.) Nevertheless, we see that the more "correct" kernel tends to have the highest power, followed by the "incorrect" kernel and then the unstructured kernel. The results suggest certain robustness against misspecification of the structure information (via treating it as prior information).

In our procedure, TOM is constructed based on the adjacency matrix *A*, and *A* is constructed based on the pairwise correlation matrix *R* as described in Appendix A when no prior network knowledge is available. We note that the adjacency matrix can also be built based on other relationship matrix. One possible choice is the partial correlation, which is known to more precisely reflect the number of edges in a network. Indeed, TOM can be replaced by other structure matrices as introduced in Dong and Horvath [63] to capture different network information besides topology overlap and connectivity. For example, the clustering coefficient, which is a density measure of local connections, can be used to weight nodes in a network with the rationale that nodes with high clustering coefficient may have large effects. Further studies are worth conducting to evaluate the performance of different choices of TOM or *A* in terms of effect assessment and to evaluate the robustness of the effect assessment with different matrix choices.

From our simulation results, we see different kernel served as the optimal choice under different network structure. Although we do not know where the causal nodes are so to select the optimal kernel in a prior, we might gain insights about the potential significant nodes based on the relative performance of the topology kernel and the connectivity kernel. Specifically, if the connectivity kernel outperforms the topology kernel, it is possible that hubs play more important roles. Otherwise, nodes with fewer connections but in the same neighborhood might deserve more attention. The results suggest the two structure kernels work in a complementary manner and we would suggest considering both in the data analysis when possible. If one really has to select one kernel method in a prior, the topology kernel may be the most appropriate choice because it consistently provides comparable or better power than the unstructured kernel method under all scenarios considered (e.g., scale-free vs. non-scale-free modules, and hub causal nodes or random causal nodes). While there are scenarios where the connectivity kernel could provide the most power improvement (such as hub causal nodes in a scale-free module), the connectivity kernel may suffer from power loss when causal nodes are non-hubs in a scale-free module (e.g., the power of the conditional test in Fig. 4).

In practice, biological pathways often share common genes, especially those that play important roles in multiple functions. When analyzing pathways with overlapping genes using the proposed framework, a potential concern is that the main effects of Module 1 and Module 2 would have collinearity and lead to unstable model fitting. Therefore when the modules are highly overlapped (i.e., the proportion of shared nodes is high in ≥1 modules), it may be better to combine the two highly overlapped modules into one, or to create a separate module for the overlapping nodes and analyze three modules (i.e., the nodes belonging uniquely to Module 1, the nodes belonging uniquely to Module 2, and overlapping nodes). On the other hand, if the magnitude of overlap is “small”, (i.e., the proportions of the shared nodes in Module 1 and in Module 2 are both low), the proposed work should still be applicable as the correlation between the two modules is low.

## Supporting Information

S1 Appendix Derivation of the score test statistics and their distributions (DOCX)

S2 Simulation Code (RAR)
