# Supplementary material for: Module-Based Association Analysis for Omics Data with Network Structure
Source: PLoS One. 2015 Mar 30;10(3):e0122309. doi: 10.1371/journal.pone.0122309 (PMC4378989; doi:10.1371/journal.pone.0122309)
Supplement: S1 Appendix — (DOCX) [file pone.0122309.s001.docx]

**Module-based Association Analysis for**

**Omics Data with Network Structure**

Zhi Wang^1^, Arnab Maity^2^, Chuhsing Kate Hsiao^3^, Deepak Voora^4^,

Rima Kaddurah-Daouk^5^, Jung-Ying Tzeng^1,2,6^

1: Bioinformatics Research Center, North Carolina State University, Raleigh NC, 27695, USA

2: Department of Statistics, North Carolina State University, Raleigh NC, 27695, USA

3: Institute of Epidemiology and Preventive Medicine, College of Public Health, National Taiwan University, Taipei, Taiwan

4: Institute for Genome Sciences and Policy, Duke University, Durham, NC, USA

5: Department of Psychiatry and Behavioral Sciences, Duke University, Durham, NC, USA

6: Department of Statistics, National Cheng-Kung University, Taiwan, R.O.C.

**RUNNING TITLE:** Module-based analysis for structured omics data

**ADDRESS FOR CORRESPONDENCE:**

Jung-Ying Tzeng, Department of Statistics and Bioinformatics Research Center,

North Carolina State University, Campus Box 7566, Raleigh NC, 27695, USA.

Tel: 919-513-2723. Fax: 919-515-7315. E-mail:jytzeng@stat.ncsu.edu.

**KEY WORDS:** module structure; network structure; association analysis; metabolomics

**APPENDIX**

**Derivation of the score test statistics and their distributions**

Consider the linear mixed model representation given in model (3). As our primary interest is to test the variance components$\tau_{1},\tau_{2},\tau_{12}$, we propose to use the restricted maximum likelihood (REML) function to estimate the variance components ($\tau_{1},\tau_{2},\tau_{12},\sigma)$. We have that the REML estimate under model (3) is

| $\mathcal{l}_{REML}\left( \tau_{1},\tau_{2},\tau_{12};Y \right)=-\left\{ \log\left\vert V \right\vert+\log\left\vert Z^{T}V^{-1}Z \right\vert+Y^{T}PY \right\}/2,$ |  |
| --- | --- |

where $V=\tau_{1}K_{1}+\tau_{2}K_{2}+\tau_{12}K_{12}+\sigma I$ is the marginal variance of Y and $P=V^{-1}-V^{-1}Z\left( Z^{T}V^{-1}Z \right)^{-1}Z^{T}V^{-1}$ is a projection matrix. The score functions based on the REML can be obtained as below (Harville 1977):

Under $H_{0}^{X_{1}*X_{2}}:\tau_{12}=0$,

| $U_{\tau_{12}}\left( \hat{\tau}_{1},\hat{\tau}_{2},0,\hat{\sigma} \right)=\left. \frac{\partial\mathcal{l}_{REML}\left( \tau_{1},\tau_{2},\tau_{12},\sigma\right)}{\partial\tau_{12}} \right\vert_{\tau_{12}=0,\tau_{1}={\hat{\tau_{1}}, \tau}_{2}=\hat{\tau_{2}}, \sigma=\hat{\sigma_{X_{1}*X_{2}}}} =\frac{1}{2}\left\{ Y^{T}P_{12}K_{12}P_{12}Y-tr\left( P_{12}K_{12} \right) \right\}.$ |  |
| --- | --- |

Under $H_{0}^{X_{1}|X_{2}}:\tau_{1}=0$ with the constraints of $\tau_{12}=0$,

| $U_{\tau_{1}}\left( 0,\hat{\tau}_{2},0,\hat{\sigma} \right)=\left. \frac{\partial\mathcal{l}_{REML}\left( \tau_{1},\tau_{2},\tau_{12},\sigma\right)}{\partial\tau_{1}} \right\vert_{\tau_{12}=0,\tau_{1}={0, \tau}_{2}=\tilde{\tau_{2}}, \sigma=\tilde{\sigma_{X1\vert X2}}}=\frac{1}{2}\left\{ Y^{T}P_{1}K_{1}P_{1}Y-tr\left( P_{1}K_{1} \right) \right\}.$ |  |
| --- | --- |

Under $H_{0}^{X_{2}|X_{1}}:\tau_{2}=0$ with the constraints of $\tau_{12}=0$,

| $U_{\tau_{2}}\left( \hat{\tau}_{1},0,0,\hat{\sigma} \right)=\left. \frac{\partial\mathcal{l}_{REML}\left( \tau_{1},\tau_{2},\tau_{12},\sigma\right)}{\partial\tau_{E}} \right\vert_{\tau_{12}=0,\tau_{1}=\tilde{\tau_{1}}{, \tau}_{2}=0, \sigma=\tilde{\sigma_{X2\vert X1}}}=\frac{1}{2}\left\{ Y^{T}P_{2}K_{2}P_{2}Y-tr\left( P_{2}K_{2} \right) \right\},$ |  |
| --- | --- |

${\mathrm{where}P}_{t}=V_{t}^{-1}-V_{t}^{-1}Z\left( Z^{T}V_{t}^{-1}Z \right)^{-1}Z^{T}V_{t}^{-1}$ for $t=\{12, 1, 2\}$,with $V_{12}=\tau_{1}K_{1}+\tau_{2}K_{2}+\sigma I, V_{1}=\tau_{2}K_{2}+\sigma I$ and $\mathrm{and} V_{2}=\tau_{1}K_{1}+\sigma I$.

**NULL DISTRIBUTION OF THE SCORE STATEISTICS FOR GE TEST**

Because score statistics are not asymptotically normal (Tzeng and Zhang 2007), we use the first term of the score statistics as the testing statistics. For interaction test, the test statistic is $T_{X_{1}*X_{2}}=\frac{1}{2}Y^{T}P_{12}K_{12}P_{12}Y.$Define $\mu=Z\beta$, then $T_{X_{1}*X_{2}}= \frac{1}{2}({Y-\mu)}^{T}P_{12}K_{12}P_{12}(Y-\mu)$ because $\mu^{T}P_{12}=0$. Further, we can rewrite $T_{X_{1}*X_{2}}=\frac{1}{2}C^{T}\left( V^{\frac{1}{2}}P_{12}K_{12}P_{12}V^{\frac{1}{2}} \right)C$, where $C=V^{-\frac{1}{2}}\left( Y-\mu\right)$and it follows a standard multivariate normal distribution. Define $e_{i}$and$\eta_{i}$ the eigenvector and eigenvalue of matrix $V^{1/2}P_{12}K_{12}P_{12}V^{1/2}/2$, respectively, then $T_{X_{1}*X_{2}}=\sum_{i=1}^{c} \eta_{i}\left( e_{i}^{T}C \right)^{2}\equiv\sum_{i=1}^{L} \eta_{i}\tilde{C}_{i}^{2}$ with $\tilde{C}_{i}^{2}$ follows a 1 $df$chi-square distribution. Therefore the distribution of $T_{X_{1}*X_{2}}$ can be approximated by the distribution of $\sum_{i=1}^{c} \hat{\eta}_{i}\chi_{i1}^{2}$, where $\hat{\eta}_{i}'s$ are the non-zero eigenvalues of $V^{\frac{1}{2}}P_{12}K_{12}P_{12}V^{\frac{1}{2}}/2\left. \right|_{\tau_{12}=0,\tau_{1}={\hat{\tau_{1}}, \tau}_{2}=\hat{\tau_{2}}, \sigma=\hat{\sigma_{X_{1}*X_{2}}}}$ . Hence, we can use a moment matching approach to obtain p-values (Duchesne and Lafaye De Micheaux 2010).

Above we use the interaction test as an example and derive the test statistics and its null distribution. By similar argument, we can approximate the null distributions of $T_{X_{1}|X_{2}}$ and $T_{X_{2}|X_{1}}$ using the distribution of $\sum_{i=1}^{c} \hat{\eta}_{i}\chi_{i1}^{2}$ where $\hat{\eta}_{i}'s$ are the non-zero eigenvalues of $V^{\frac{1}{2}}P_{1}K_{1}P_{1}V^{\frac{1}{2}}/2\left. \right|_{\tau_{12}=0,\tau_{1}={0, \tau}_{2}=\tilde{\tau_{2}}, \sigma=\tilde{\sigma_{X_{1}|X_{2}}}}$and$V^{\frac{1}{2}}P_{2}K_{2}P_{2}V^{\frac{1}{2}}/2\left. \right|_{\tau_{12}=0,\tau_{1}=\tilde{\tau_{1}}{, \tau}_{2}=0, \sigma=\tilde{\sigma_{X_{2}|X_{1}}}}$, respectively.

**EM ALGORITHM FOR THE REML ESTIMATES OF** $\boldsymbol{\tau}_{\boldsymbol{1}}$ **AND** $\boldsymbol{\tau}_{\boldsymbol{2}}$ **WHEN TESTING** $\boldsymbol{H}_{\boldsymbol{0}}^{\boldsymbol{X}_{\boldsymbol{1}}\boldsymbol{*}\boldsymbol{X}_{\boldsymbol{2}}}\boldsymbol{:}\boldsymbol{\tau}_{\boldsymbol{12}}\boldsymbol{=0}$

Using the interaction test ($T_{X_{1}*X_{2}})$as an example, we derive the EM algorithm for estimating the nuisance variance components (VC), $\tau_{1}, \tau_{2}, \mathrm{and}\sigma$, under $H_{0}^{X_{1}*X_{2}}$. The EM algorithms for estimating nuisance VCs for the $X_{1}|X_{2}$ test and the $X_{2}|X_{1}$ test can be obtained by zeroing out the corresponding variance components. In short, the derivation of the EM algorithm is similar to the one derived in Tzeng et al. (2011). Let $u=A^{T}Y$ with $A^{T}A=I_{n*n} and AA^{T}=I-Z\left( Z^{T}Z \right)^{-1}{ZX}^{T}$. Then $f(u|h_{1},h_{2})$ follows normal distribution with mean $A^{T}h_{1}+A^{T}h_{2}$ and variance $\sigma I$ and does not depend on the fixed effect $\beta$. Therefore, the REML estimators of $\tau_{1}$ and $\tau_{2}$ can be based on their marginal distributions, $f\left( u \right)=\int\int f\left( u | h_{1},h_{2} \right)f\left( h_{1},h_{2} \right)dh_{1}dh_{2}$. This motivated the EM algorithm based on observed data $u$ and missing data $h_{1}$ and $h_{2}$.

The complete data log likelihood is given be

$$logf\left( u,h_{1},h_{2};\tau_{1},\tau_{2},\sigma\right)=logf\left( u | h_{1},h_{2};\tau_{1},\tau_{2},\sigma\right)+logf\left( h_{2};\tau_{2},\sigma\right)+logf\left( h_{1};\tau_{1},\sigma\right)$$

$$=-\frac{n-d}{2}\log\sigma-\frac{1}{2\sigma}\left( u-A^{T}h_{1}-A^{T}h_{2} \right)^{T}\left( u-A^{T}h_{1}-A^{T}h_{2} \right)$$

$$-\frac{n}{2}\log\tau_{1}-\frac{1}{2}\log\left| K_{1} \right|-\frac{1}{2\tau_{1}}h_{1}^{T}K_{1}^{-1}h_{1}$$

$-\frac{n}{2}\log\tau_{2}-\frac{1}{2}\log\left| K_{2} \right|-\frac{1}{2\tau_{2}}h_{2}^{T}K_{2}^{-1}h_{2}$.

In the expectation step, we calculate the expected value of the log likelihood function, $Q(\tau_{1},\tau_{2},\sigma|\hat{\tau}_{1}^{\left( t \right)},\hat{\tau}_{2}^{\left( t \right)}, \hat{\sigma}^{(t)})$ with respect to the observed data $u$ under the current (the $t$-th iteration) estimate of the parameters $\hat{\tau}_{1}^{(t)},\hat{\tau}_{2}^{(t)}and \hat{\sigma}^{(t)},$

$Q\left( \tau_{1},\tau_{2},\sigma| \hat{\tau}_{1}^{\left( t \right)},\hat{\tau}_{2}^{\left( t \right)}, \hat{\sigma}^{\left( t \right)} \right)=E\left[ logf\left( u,h_{1},h_{2};\tau_{1},\tau_{2},\sigma\right)|u;\hat{\tau}_{1}^{\left( t \right)},\hat{\tau}_{2}^{\left( t \right)}, \hat{\sigma}^{\left( t \right)} \right]$

$$=-\frac{n-d}{2}\log\sigma-\frac{1}{2\sigma}E\left\{ \left( u-A^{T}h_{1}-A^{T}h_{2} \right)^{T}\left( u-A^{T}h_{1}-A^{T}h_{2} \right)|u;\hat{\tau}_{1}^{\left( t \right)},\hat{\tau}_{2}^{\left( t \right)}, \hat{\sigma}^{\left( t \right)} \right\}$$

$$-\frac{n}{2}\log\tau_{1}-\frac{1}{2}\log\left| K_{1} \right|-\frac{1}{2\tau_{G}}E\left\{ h_{1}^{T}K_{1}^{-1}h_{1}|u;\hat{\tau}_{1}^{\left( t \right)},\hat{\tau}_{2}^{\left( t \right)}, \hat{\sigma}^{\left( t \right)} \right\}$$

$$-\frac{n}{2}\log\tau_{2}-\frac{1}{2}\log\left| K_{2} \right|-\frac{1}{2\tau_{2}}E\left\{ h_{2}^{T}K_{2}^{-1}h_{2}|u;\hat{\tau}_{1}^{\left( t \right)},\hat{\tau}_{2}^{\left( t \right)}, \hat{\sigma}^{\left( t \right)} \right\}.$$

In the maximization step, we maximize $Q(\tau_{1},\tau_{2},\sigma|\hat{\tau}_{1}^{\left( t \right)},\hat{\tau}_{2}^{\left( t \right)}, \hat{\sigma}^{(t)})$ by solving $\frac{\partial Q}{\partial\tau_{1}}=0, \frac{\partial Q}{\partial\tau_{2}}=0 \mathrm{and}\frac{\partial Q}{\partial\sigma}=0$ and obtain the following estimates

$$\hat{\tau}_{1}^{\left( t+1 \right)}=\frac{1}{n}E\left\{ h_{1}^{T}K_{1}^{-1}h_{1}|u;\hat{\tau}_{1}^{\left( t \right)},\hat{\tau}_{2}^{\left( t \right)}, \hat{\sigma}^{\left( t \right)} \right\}$$

$$=\frac{1}{n}\left\{ \hat{\tau}_{1}Y^{T}P_{12}K_{1}P_{12}Y+tr(\tau_{1}I-\tau_{1}^{2}P_{12}K_{1}) \right\};$$

$$\hat{\tau}_{2}^{\left( t+1 \right)}=\frac{1}{n}E\left\{ h_{2}^{T}K_{2}^{-1}h_{2}|u;\hat{\tau}_{1}^{\left( t \right)},\hat{\tau}_{2}^{\left( t \right)}, \hat{\sigma}^{\left( t \right)} \right\}$$

$$=\frac{1}{n}\left\{ \hat{\tau}_{2}Y^{T}P_{12}K_{2}P_{12}Y+tr(\tau_{2}I-\tau_{2}^{2}P_{12}K_{2}) \right\};$$

$$\hat{\sigma}^{\left( t+1 \right)}=\frac{1}{n-d}E\left\{ \left( u-A^{T}h_{1}-A^{T}h_{2} \right)^{T}\left( u-A^{T}h_{1}-A^{T}h_{2} \right)|u;\hat{\tau}_{1}^{\left( t \right)},\hat{\tau}_{2}^{\left( t \right)}, \hat{\sigma}^{\left( t \right)} \right\}$$

$$=\left( Y-\tilde{M} \right)^{T}AA^{T}\left( Y-\tilde{M} \right)+tr\left( A^{T}\tilde{V}A \right),$$

where$AA^{T}=I-Z\left( Z^{T}Z \right)^{-1}Z^{T}$,$\tilde{M}=E\left( h_{1}+h_{2} | u;\hat{\tau}_{1}^{\left( t \right)},\hat{\tau}_{2}^{\left( t \right)}, \hat{\sigma}^{\left( t \right)} \right)=\left( \tau_{1}K_{1}+\tau_{2}K_{2} \right)P_{12}$,$\tilde{V}=var\left( h_{1}+h_{2} | u;\hat{\tau}_{1}^{\left( t \right)},\hat{\tau}_{2}^{\left( t \right)}, \hat{\sigma}^{\left( t \right)} \right)=\tau_{1}K_{1}-\tau_{1}^{2}K_{1}P_{12}K_{1}+\tau_{2}K_{2}-\tau_{2}^{2}K_{2}P_{12}K_{2}-2\tau_{1}\tau_{2}K_{2}P_{12}K_{1},$and $\tilde{M}$ and $\tilde{V}$ are obtained from the joint distribution of $(u,h_{1},h_{2})$.

**REFERENCE**

Duchesne P, Lafaye De Micheaux P. 2010. Computing the distribution of quadratic forms: Further comparisons between the Liu–Tang–Zhang approximation and exact methods. *Comput Stat Data Anal* **54**: 858-862.

Harville D. 1977. Maximum likelihood approaches to variance component estimation and related problems. *J Am Stat Assoc* **72**:322–340.

Tzeng JY, Zhang D. (2007) Haplotype-based association analysis via variance-components score test. *Am J Hum Genet* **81**:927-38.

Tzeng JY, Zhang D, Pongpanich M, Smith C, McCarthy MI, Sale MM, Worrall BB, Hsu FC, Thomas DC, Sullivan PF. 2011. Studying gene and gene-environment effects of uncommon and common variants on continuous traits: a marker-set approach using gene-trait similarity regression. *Am J Hum Genet* **12**: 277-88.

Zhang B, Horvath S. 2005. A general framework for weighted gene co-expression network analysis. *Stat Appl Genet Molec Biol* **4**: 1128.
